# Artepillin C, a Typical Brazilian Propolis-Derived Component, Induces Brown-Like Adipocyte Formation in C3H10T1/2 Cells, Primary Inguinal White Adipose Tissue-Derived Adipocytes, and Mice

**DOI:** 10.1371/journal.pone.0162512

**Published:** 2016-09-06

**Authors:** Sho Nishikawa, Hiroki Aoyama, Misa Kamiya, Jun Higuchi, Aiko Kato, Minoru Soga, Taeko Kawai, Kazuki Yoshimura, Shigenori Kumazawa, Takanori Tsuda

**Affiliations:** 1 College of Bioscience and Biotechnology, Chubu University, Kasugai, Aichi 487-8501, Japan; 2 Department of Food and Nutritional Sciences, University of Shizuoka, Suruga-ku, Shizuoka, Shizuoka 422-8526, Japan; Brown University Warren Alpert Medical School, UNITED STATES

## Abstract

Induction of brown-like adipocytes (beige/brite cells) in white adipose tissue (WAT) suggests a new approach for preventing and treating obesity via induction of thermogenesis associated with uncoupling protein 1 (UCP1). However, whether diet-derived factors can directly induce browning of white adipocytes has not been well established. In addition, the underlying mechanism of induction of brown-like adipocytes by diet-derived factors has been unclear. Here, we demonstrate that artepillin C (ArtC), which is a typical Brazilian propolis-derived component, significantly induces brown-like adipocytes in murine C3H10T1/2 cells and primary inguinal WAT (iWAT)-derived adipocytes. This significant induction is due to activation of peroxisome proliferator-activated receptor γ and stabilization of PRD1-BF-1-RIZ1 homologous domain-containing protein-16 (PRDM16). Furthermore, the oral administration of ArtC (10 mg/kg) for 4 weeks significantly induced brown-like adipocytes accompanied by significant expression of UCP1 and PRDM16 proteins in iWAT of mice, and was independent of the β3-adrenergic signaling pathway via the sympathetic nervous system. These findings may provide insight into browning of white adipocytes including the molecular mechanism mediated by dietary factors and demonstrate that ArtC has a novel biological function with regard to increasing energy expenditure by browning of white adipocytes.

## Introduction

One of the most serious public health problems in recent times has been obesity, which continues to increase globally. Mammals possess two types of adipose tissue, white and brown adipose tissue (WAT and BAT, respectively), which have physiologically distinct functions: WAT stores excess energy as triglycerides and BAT dissipates excess energy through heat production and can thereby suppress body weight gain and metabolic disease [1]. Thermogenesis in BAT requires a high mitochondrial content and the expression of uncoupling protein 1 (UCP1), which causes mitochondrial proton leak, leading to the generation of heat instead of the production of ATP [2]. Recent studies using ^18^fluoro-labeled 2-deoxyglucose positron emission tomography have shown the presence of functional BAT deposits in adult humans [3–6]. Induction of thermogenesis in BAT may be a potential therapeutic intervention against obesity and the related metabolic disorders [7, 8].

Current research has established two types of brown adipocytes, which have distinct developmental origins. "Classical" brown adipocytes in the interscapular and other peripheral tissues develop from myoblastic-like Myf5-positive precursors [9]. By contrast, "inducible" brown adipocytes (brown-like adipocytes, also known as "beige" or "brite" cells) develop from Myf5-negative precursors during the postnatal stage and appear to be interspersed throughout WAT. Brown-like adipocytes possess many of the biochemical and morphological characteristics of classical brown adipocytes [10]. “Inducible” refers to a characteristic of the cells, where development can be induced in response to chronic cold or β3-adrenergic receptor (β3-AR) agonists [2, 11]. Therefore, brown-like adipocytes have the ability to dissipate excess energy via heat production, and are likely a promising therapeutic target for the treatment of obesity and its related disorders.

In response to these results, several previous clinical trials were launched to treat obesity using β3-AR agonists. However, these trials yield very low clinical efficacy, and there were several side effects [12]. It has been shown that activation of full peroxisome proliferator-activated receptor (PPAR) γ agonists, such as rosiglitazone (thiazolidinedione compound) used as an antidiabetic drug, induces brown-like adipocytes [13]. However, thiazolidinedione-type drugs also act as strong PPARγ ligands in white adipocytes, and cause undesirable side effect such as body fat accumulation and edema [14].

Recently, the search for dietary factors that may be beneficial in the induction of brown-like adipocyte proliferation has received considerable attention. Although induction of brown-like adipocytes by dietary factors is thought to be preferable for prevention and treatment of obesity and its related diseases as a new strategy, there has been little evidence that dietary factors can directly induce brown-like adipocytes which are responsible for anti-obesity via thermogenesis.

**Fig 1:**
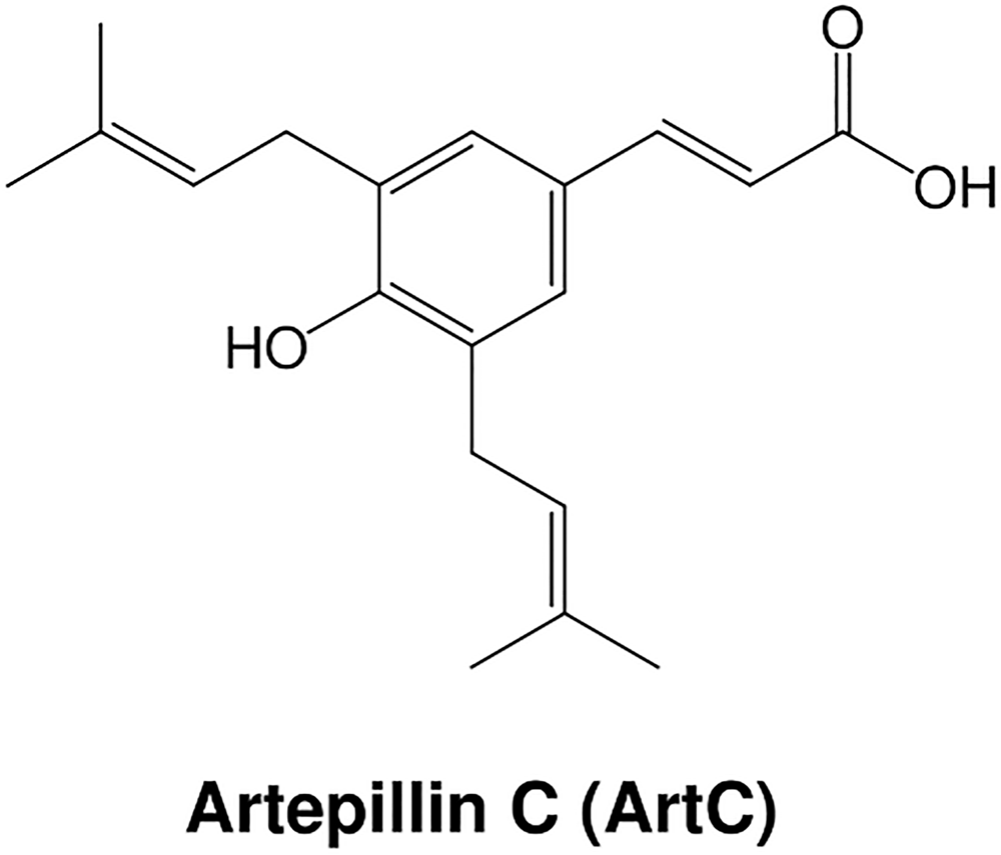
Chemical structure of ArtC.

In the present study, we have demonstrated that artepillin C (ArtC) (Fig 1), which is a typical component of Brazilian propolis (a resinous plant-based material collected by honeybees and used in hive construction) [15–17], significantly induced brown-like adipocyte formation via its action as a PPARγ agonist and via stabilization of PRD1-BF-1-RIZ1 homologous domain-containing protein-16 (PRDM16), which is required for the development of brown-like adipocytes [18, 19]. In addition, we have demonstrated that oral administration of ArtC significantly induced browning accompanied by upregulation of UCP1 protein expression in inguinal WAT (iWAT) in C57BL/6J mice.

## Materials and Methods

### Chemicals

The purity of all chemicals was >98%. ArtC was isolated and purified from Brazilian propolis [16, 17]. In brief, propolis originating from *Baccharis dracunculifolia* was collected in Minas Gerais, Brazil in 2008, and stored at 4°C until use. The propolis sample was extracted with methanol, and pure ArtC was isolated from the extract by repeated column chromatography. The purity of the compound was >99% as determined from its ^1^H-NMR spectrum. Dexamethasone, 3-isobutyl-1-methylxanthine (IBMX), triiodothyronine (T3), and cycloheximide were obtained from Wako Pure Chemical Industries (Osaka, Japan). Rabbit polyclonal β-actin antibody (#4967) and rabbit polyclonal cytochrome c oxidase subunit IV (CoxIV) antibody (#4844) were obtained from Cell Signaling Technology (Beverly, MA). Rabbit polyclonal UCP1 antibody (ab10983) and rabbit polyclonal PRDM16 antibody (ab106410) were obtained from Abcam (Tokyo, Japan).

### Cell culture (C3H10T1/2 cells)

The murine C3H10T1/2 cell line (IFO050415, Health Science Research Resources Bank, Osaka, Japan) was a gift (April, 2013) from Dr. Hitoshi Yamashita, Chubu University, Japan. This cell line is a stem cell that can be differentiated to brown-like adipocytes [20]. Cells were cultured in Dulbecco’s modified Eagle’s medium (DMEM) supplemented with 10% fetal bovine serum (FBS) at 37°C in a humidified atmosphere with 5% CO_2_. Confluent cells were placed in a differentiation medium (DMEM containing 10% FBS, 1 μM dexamethasone, 0.5 mM IBMX, 3 nM T3, and 2 μM insulin) with or without various compounds for 2 days before the medium was changed to induction medium (DMEM containing 10% FBS, 3 nM T3, and 2 μM insulin) with or without various compounds. The medium was changed every 2 days and the cells were cultured for an additional 6 days to obtain mature adipocytes.

### Isolation of the stromal vascular fraction (SVF) from iWAT and primary adipocyte culture

Male C57BL/6J mice (7–9 weeks of age) were obtained from Japan SLC (Shizuoka, Japan) and housed in an animal room (23 ± 3°C, 12 h light:dark cycle, lights on from 8:00 to 20:00 h) and allowed ad libitum access to water and a standard laboratory diet (CE-2, CLEA Japan, Tokyo, Japan) [21]. After 1 week of breeding, mice were killed and iWAT was carefully removed and the SVF was isolated [22]. Briefly, isolated iWAT was minced and suspended and digested in 0.2% collagenase (125 collagen digestion units/mL, C6885, Sigma-Aldrich Japan, Tokyo, Japan) solution at 37°C for 30 min. After digestion, the cell suspension was filtered using a cell strainer and centrifuged. The obtained pellet (SVF) was re-suspended in DMEM supplemented with 10% FBS, and plated on collagen-coated dishes (CORNING, NY). Confluent cells were differentiated and induced to adipocytes as well as C3H10T1/2 cells. The experimental design was approved by the Animal Experiment Committee, Chubu University, and the mice were maintained in accordance with their guidelines (Permission No. 2610031 and 2710011).

### Analysis of mRNA levels

At 8 days (C3H10T1/2 cells) or 12 days (primary adipocyte) after the differentiation and induction of preadipocytes to adipocytes, total adipocyte RNA was isolated using QIAzol TM reagent (QIAGEN, Tokyo, Japan) according to the manufacturer’s directions. As in our previous studies [23, 24], total RNA (1.0 μg) was reverse transcribed to cDNA and gene expression was quantified with a real-time PCR system (ABI PRISM 7300 Sequence Detection System; Life Technologies, Tokyo, Japan). The assay identification numbers of the TaqMan gene expression assays were: UCP1, Mm01244861_m1; TATA box binding protein (TBP), Mm00446971_m1; cell death-inducing DNA fragmentation factor, alpha subunit-like effector A (Cidea), Mm00432554_m1; cytochrome C oxidase subunit VIII b (Cox8b), Mm00432648_m1; elongation of very long chain fatty acids-like 3 (Elovl3), Mm00468164_m1; and β3-AR, Mm02601819_g1. The gene expression level was expressed relative to the control (= 1.0) after normalization using the TBP gene expression level.

### Immunoblot analysis of various proteins

After various treatments or incubations, the cells were lysed [25]. Aliquots of the supernatant were treated with Laemmli sample buffer for 5 min at 100°C [26]. As in our previous study [21], samples (50 μg protein) were loaded into an SDS-PAGE system and resulting gels were transblotted onto PVDF membranes before the sheets were probed with various antibodies for 16 h at 4°C, and reacted with horseradish peroxidase-conjugated anti-rabbit or anti-mouse antibody. Immunoreactivity was then visualized using Pierce Western Blotting Substrate (Thermo Fisher Scientific, Yokohama, Japan) [21]. All experiments were performed in triplicate, and representative results are shown.

### PRDM16 protein stability

C3H10T1/2 cells were differentiated into mature adipocytes in the presence or absence of ArtC as described above. At 8 days after differentiation, the cells were incubated in medium containing 20 μg/mL cycloheximide and harvested at different time points and under various conditions, as indicated [27]. After the indicated period, the cells were lysed and immunoblot analysis for PRDM16 protein was performed as described above.

### Animal experiments

C57BL/6J mice (male, 4 weeks of age) were obtained from Japan SLC. The mice were housed in an animal room (23 ± 3°C, 12 h light:dark cycle, lights on from 8:00 to 20:00 h) and allowed ad libitum access to water and a standard laboratory diet (CE-2) [21]. After 1 week of breeding, mice were divided into three groups: control (vehicle, 5% DMSO–4% Tween 80 in water), low-dose (5 mg/kg) and high-dose (10 mg/kg) ArtC. Vehicle or ArtC was orally administered to mice by direct stomach intubation every day for 4 weeks. Then, the mice were killed and blood samples were collected. iWAT, BAT, and epididymal WAT (eWAT) were removed and weighed. Small pieces of adipose tissues were then fixed and used for histological analysis. Aliquots of tissues were homogenized and the mitochondrial fraction was isolated using the method of Lau et al. [28], and immunoblot analysis was performed as described above. The experimental design was approved by the Animal Experiment Committee, Chubu University, and the mice were maintained in accordance with their guidelines (Permission No. 2710011).

### Hematoxylin & eosin (H&E) staining and immunostaining

Adipose tissues were fixed in 4% paraformaldehyde for 24 h at 4°C. Then the tissues were embedded in paraffin and cut into slices for UCP1 (5-μm thick) and PRDM16 (3-μm thick) immunostaining, and deparaffinized and rehydrated using xylene, ethanol, and water. Adipose tissue sections were stained with H&E (both from Sakura Finetek Japan, Tokyo, Japan) or UCP1 and PRDM16 immunostained. For immunohistochemistry, slides were submerged in 0.1 M sodium citrate (pH 6.0) and heated to 100°C for 15 min in a laboratory microwave (500 W). Slides were incubated with 0.3% hydrogen peroxide for 30 min to block endogenous peroxidase, and 2.5% normal horse serum (included in kit) for 20 min at room temperature. Slides were then incubated with anti-UCP1 antibody (1:500) and anti-PRDM16 antibody (1:150) for 16 h at 4°C, and with anti-rabbit IgG (included in kit) for 30 min at room temperature. Labeling was visualized with 3, 3′-diaminobenzidine (DAB) as the chromogen using ImmPress kit and Immpact DAB peroxidase substrate kit (Vector Laboratories, Burlingame, CA) according to the manufacturer’s directions. Then, sections were counterstained with hematoxylin.

### Measurement of plasma norepinephrine (NE) concentration

Plasma NE concentration was measured using an ELISA specific for NE (ELISA kit, Cloud-Clone Corp., TX) according to the manufacturer’s instructions.

### Statistical analysis

All data are expressed as the means ± SEM. Differences between means were compared by the Tukey-Kramer test or Student’s *t*-test. For all statistical tests, differences with *P* values < 0.05 were considered significant.

## Results

### ArtC induces brown-like adipocyte formation in C3H10T1/2 cells and primary iWAT-derived adipocytes

**Fig 2:**
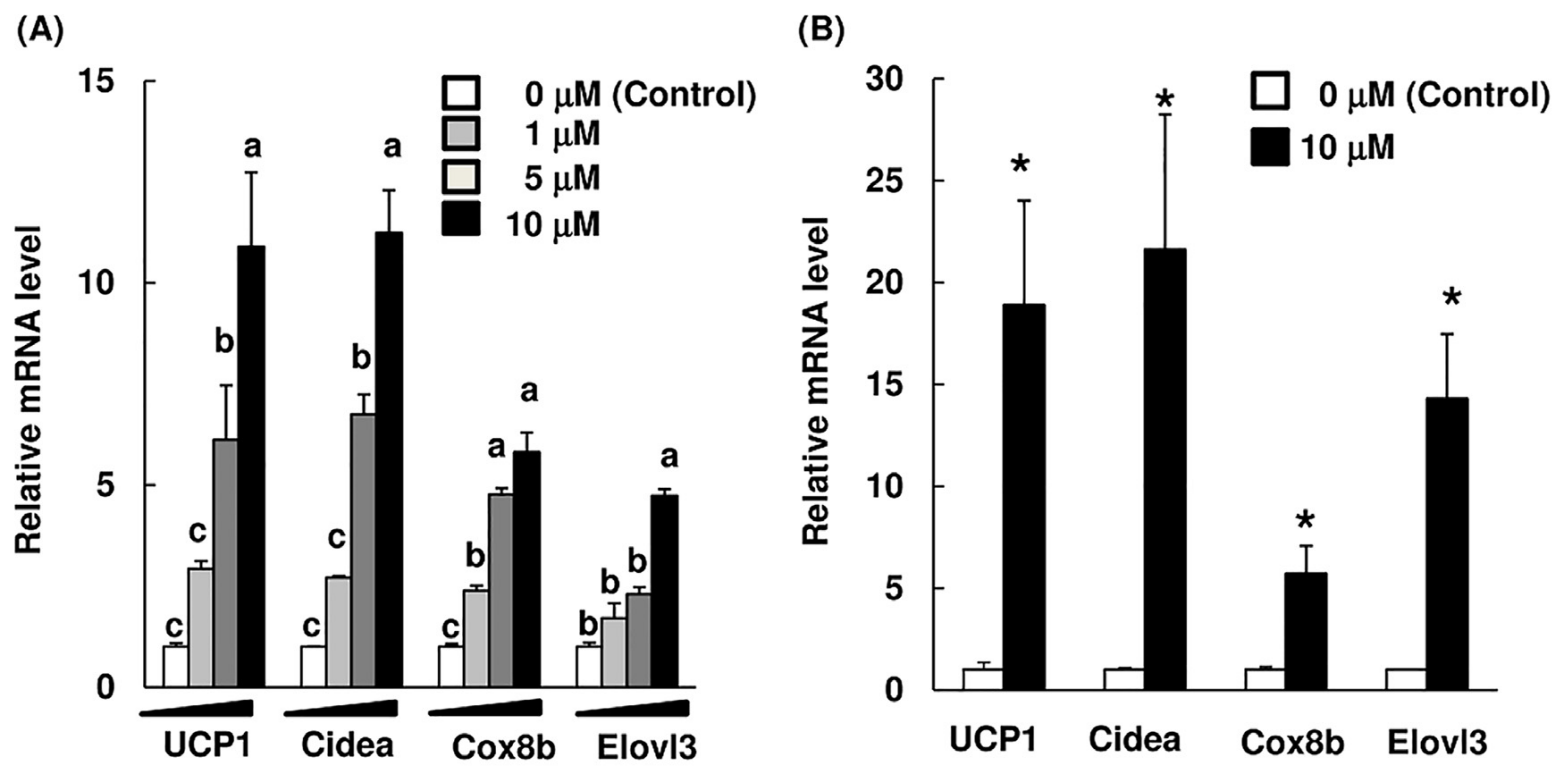
mRNA levels of brown-like adipocyte markers in C3H10T1/2 cells and primary iWAT-derived adipocytes treated with ArtC or vehicle (control). C3H10T1/2 cells (A) and primary iWAT-derived adipocytes (B). The mRNA levels are expressed as fold-change relative to control (= 1) after normalization using the expression levels of the TBP gene. All data are shown as means ± SEM (*n* = 3). (A) Values without a common letter are significantly different at *P* < 0.05 (Tukey-Kramer test); (B) * mean values are significantly different from those of the control at *P* < 0.05 (Student’s *t*-test).

We first examined whether ArtC induced brown-like adipocytes using two cellular systems. C3H10T1/2 cells were differentiated into adipocytes in the presence or absence of ArtC, and the mRNA level of brown-like adipocytes markers was assayed. Administration of ArtC significantly increased the mRNA levels of UCP1, Cidea, Cox8b, and Elovl3 in a dose-dependent manner (Fig 2A). The use of primary adipocytes derived from the SVF of isolated iWAT is a suitable evaluation method for inducing the browning of white adipocytes in response to drugs or food-derived factors [22]; likewise, using C3H10T1/2 cells is a suitable method for inducing development of brown-like adipocytes. Administration of ArtC during the differentiation significantly induced the brown-like adipocytes implicated-gene expression levels in primary iWAT-derived adipocytes (Fig 2B).

### ArtC induces brown-like adipocyte formation via its action as a PPARγ agonist and stabilization of PRDM16

**Fig 3:**
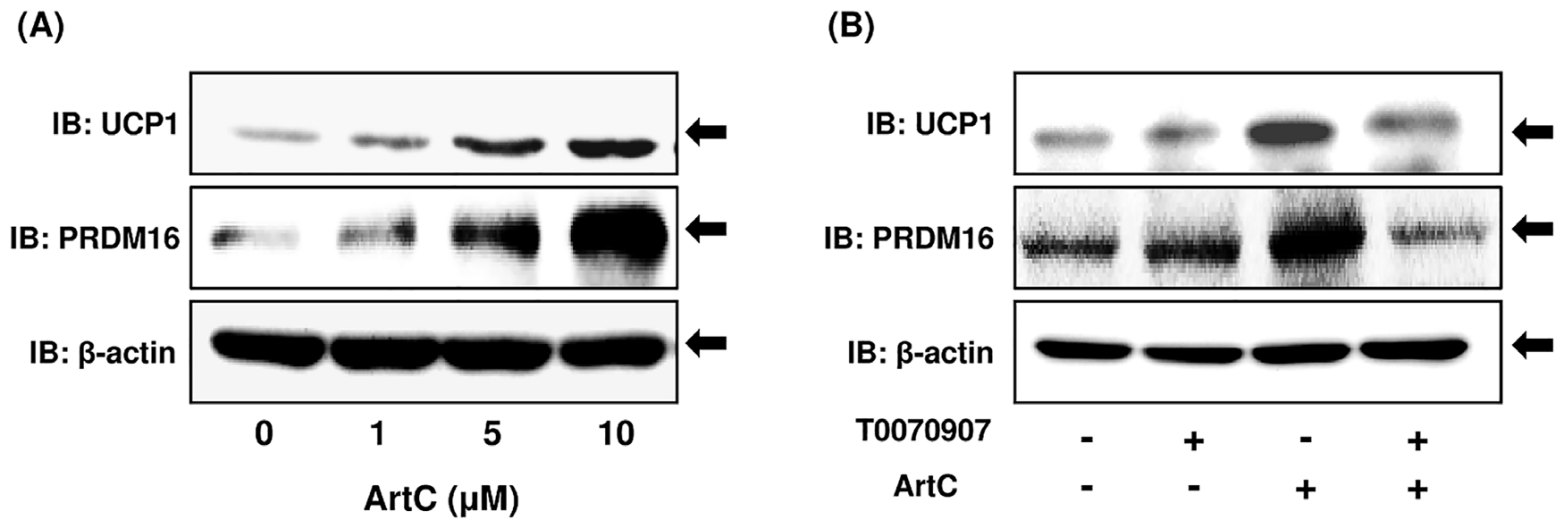
Immunoblot analysis of UCP1, PRDM16, and β-actin proteins in C3H10T1/2 cells differentiated with vehicle or ArtC. (A) the expression of UCP1, PRDM16, and β-actin proteins in C3H10T1/2 cells differentiated with ArtC at different doses; (B) the effect of the PPARγ antagonist T0070907 on ArtC-induced UCP1 and PRDM16 protein expression.

**Fig 4:**
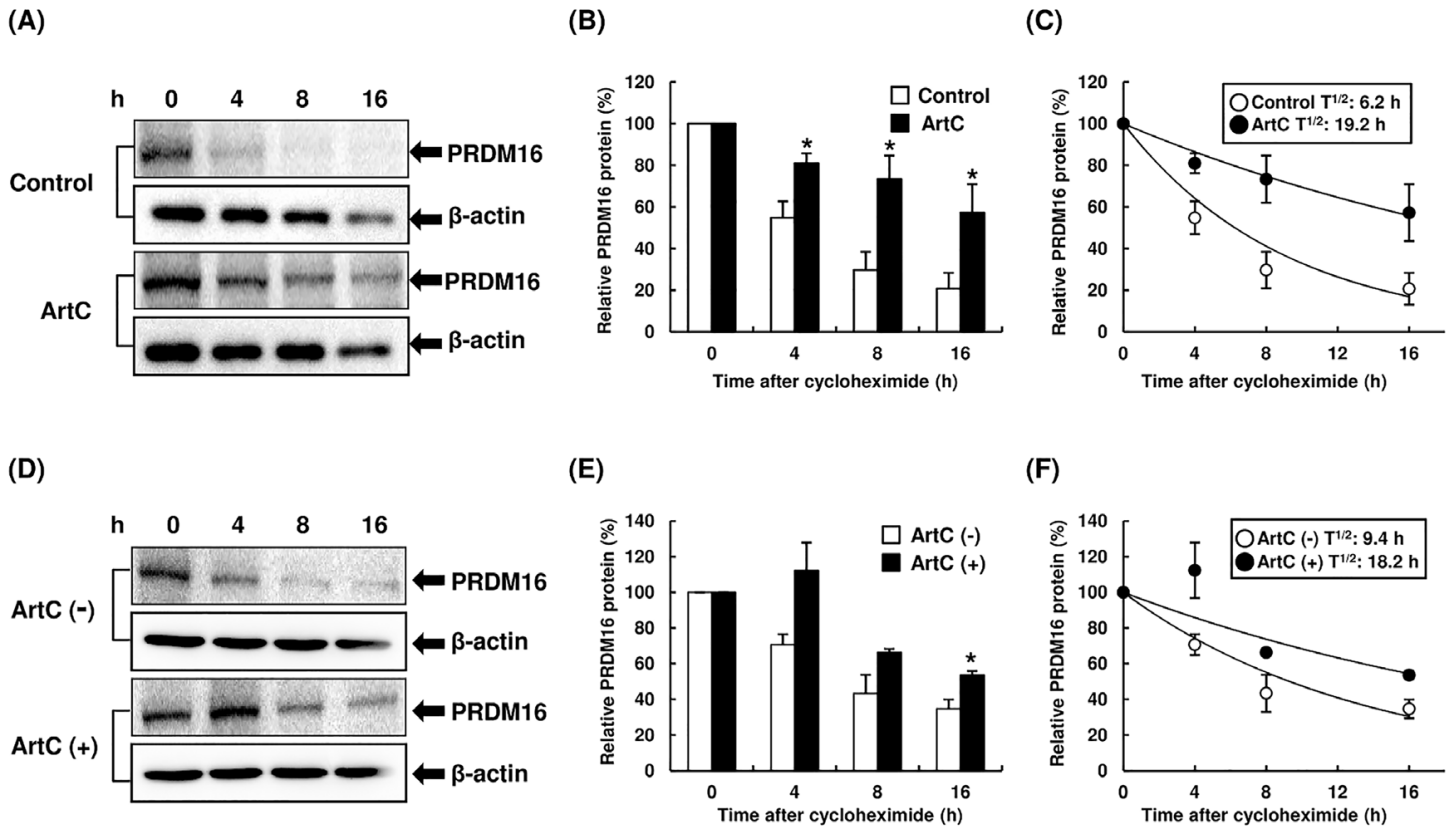
The degradation of PRDM16 protein levels in C3H10T1/2 cells treated with vehicle or ArtC. (A, B) The degradation of PRDM16 protein in C3H10T1/2 cells differentiated with or without ArtC. C3H10T1/2 cells were differentiated with or without ArtC for 8 days, and then cells were treated with cycloheximide and the degradation of PRDM16 protein was examined using immunoblot analysis at the indicated time point. (C) PRDM16 protein stability over time, plotted from data in panel (B). (D, E) The degradation of PRDM16 protein in C3H10T1/2 cells differentiated without ArtC. C3H10T1/2 cells were differentiated without ArtC for 8 days, then the cells were treated with vehicle or ArtC in medium containing cycloheximide. The degradation of PRDM16 protein was examined using immunoblot analysis at the indicated time point. (F) PRDM16 protein stability over time, plotted from data in panel (E). (B, E) The data are presented as means ± SEM (*n* = 3–5); * mean values are significantly different from those of the control (B) or ArtC (-) (E) at *P* < 0.05 (Student’s *t*-test).

Next, we confirmed that ArtC induces UCP1 and PRDM16 proteins, and functions as an inducer of brown-like adipocytes via its action as a PPARγ agonist. The administration of ArtC clearly induced UCP1 and PRDM16 proteins in a concentration-dependent manner (Fig 3A). Furthermore, the administration of T0070907 (a selective PPARγ antagonist) during differentiation resulted in inhibition of ArtC-induced UCP1 and PRDM16 protein expression (Fig 3B). Recent studies showed that rosiglitazone induced browning of the white adipocytes via not only activation of PPARγ but also stabilization and accumulation of the PRDM16 protein [27], and the mechanism of ArtC-induced brown-like adipocyte formation may be similar to that of rosiglitazone. Therefore, we examined the effect of PRDM16 protein stabilization on ArtC-induced brown-like adipocytes. C3H10T1/2 cells were differentiated with or without ArtC for 8 days, and then the cells were treated with cycloheximide and the degradation of PRDM16 protein was examined. ArtC clearly inhibited degradation of PRDM16 protein and induced stabilization (Fig 4A and 4B). In addition, ArtC extended the half-life of PRDM16 protein from 6.2 to 19.2 h (Fig 4C). Next, C3H10T1/2 cells were differentiated without ArtC for 8 days, and then the cells were treated with vehicle or ArtC. Treatment of the cells with ArtC also induced significant stabilization, accumulation of the PRDM16 protein compared to vehicle (Fig 4D and 4E), and extended the half-life of PRDM16 protein from 9.4 to 18.2 h (Fig 4F).

### Administration of ArtC induces brown-like adipocyte formation in iWAT

**Fig 5:**
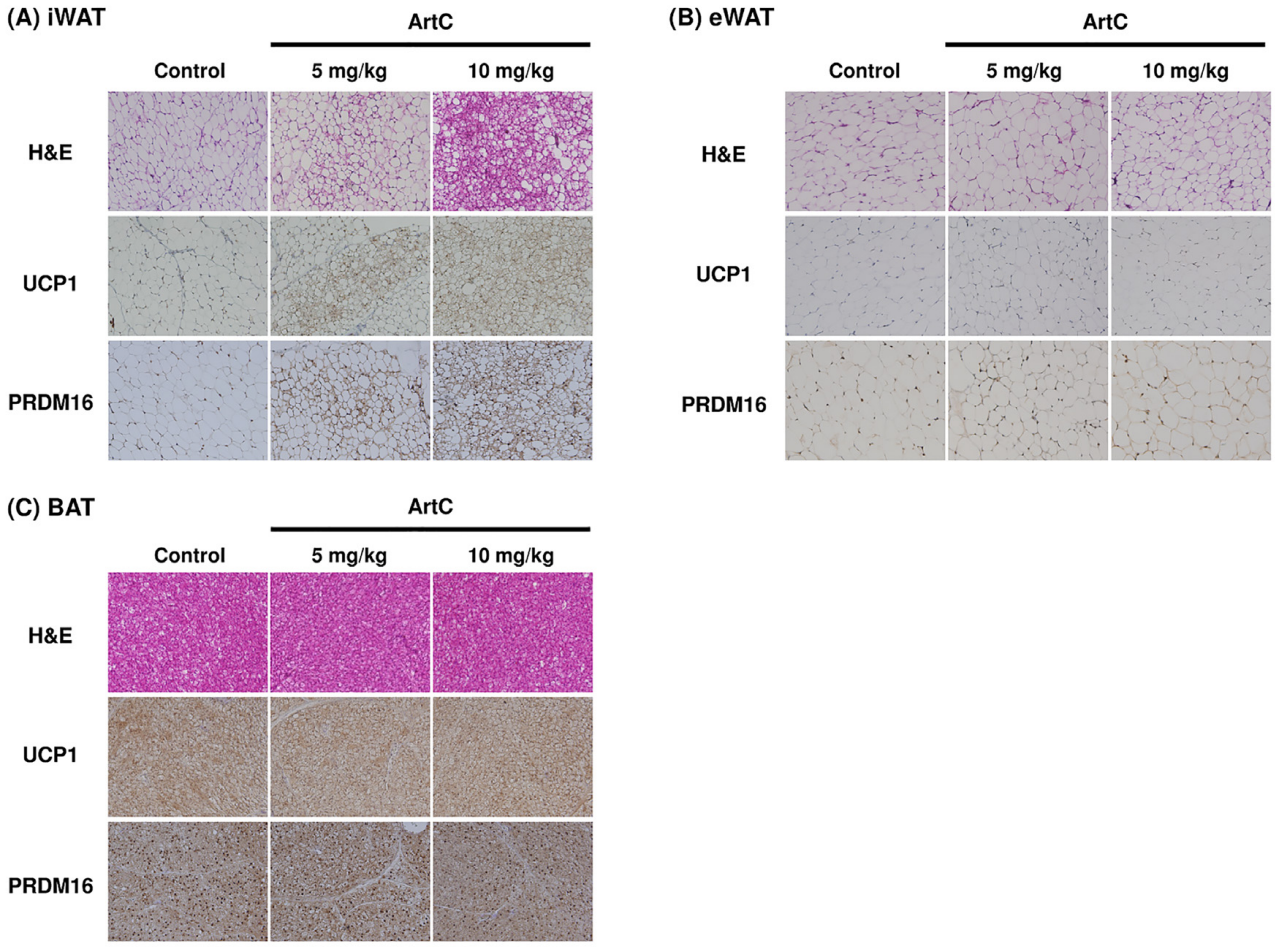
Representative images of H&E staining and immunohistochemical staining of UCP1 and PRDM16 in sections of various adipose tissues from mice treated with vehicle (control) or ArtC (5 mg/kg or 10 mg/kg) for 4 weeks. (A) iWAT, (B) eWAT, (C) BAT.

**Fig 6:**
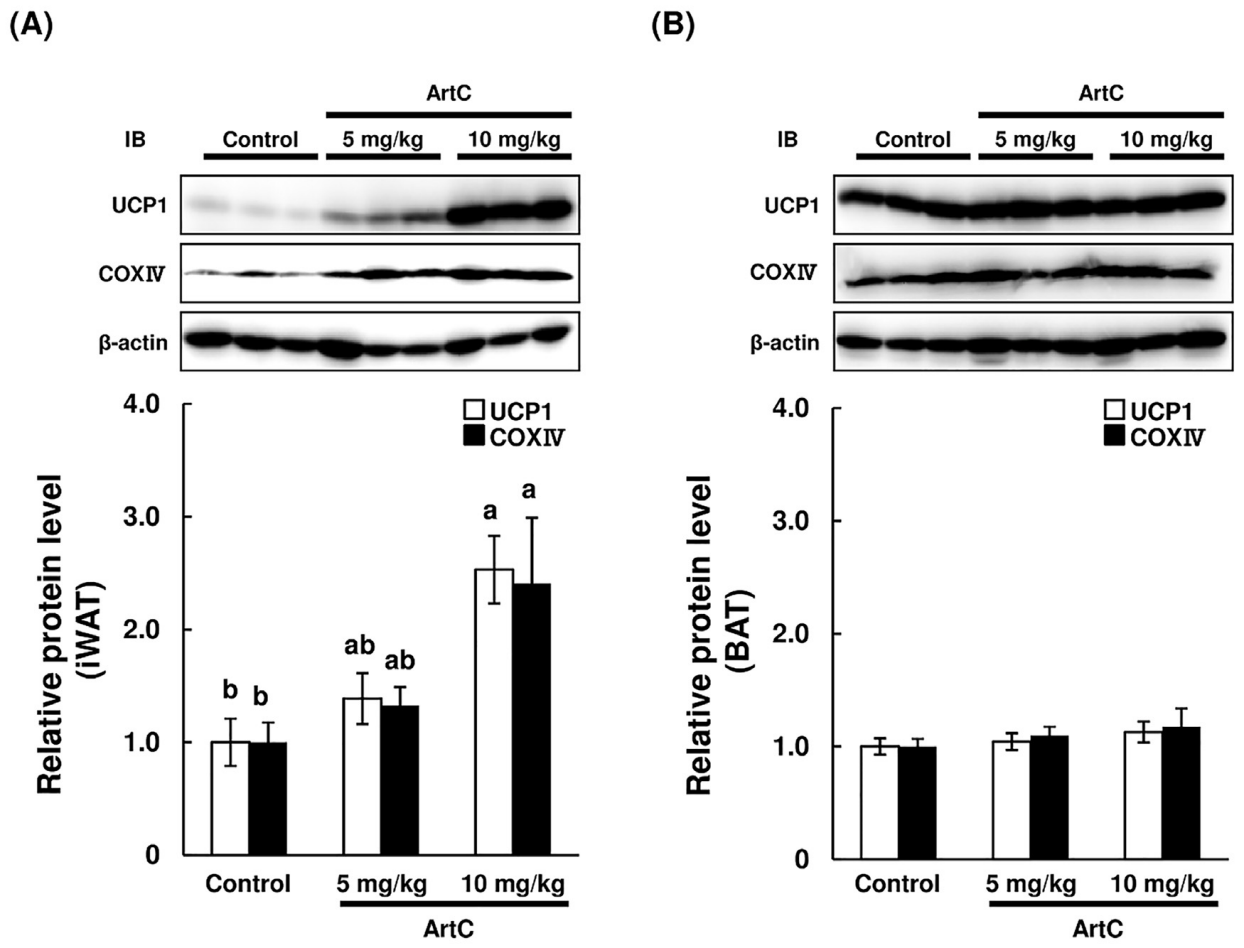
Immunoblot analysis of UCP1, CoxIV, and β-actin in the iWAT and BAT of mice treated with vehicle (control) or ArtC (5 mg/kg or 10 mg/kg) for 4 weeks. (A) iWAT, (B) BAT. Relative protein levels are expressed as fold-change relative to control (= 1) after normalization using the β-actin protein level. Data are presented as means ± SEM (*n* = 9–10). Mean values without a common letter are significantly different at *P* < 0.05(Tukey-Kramer test).

**Fig 7:**
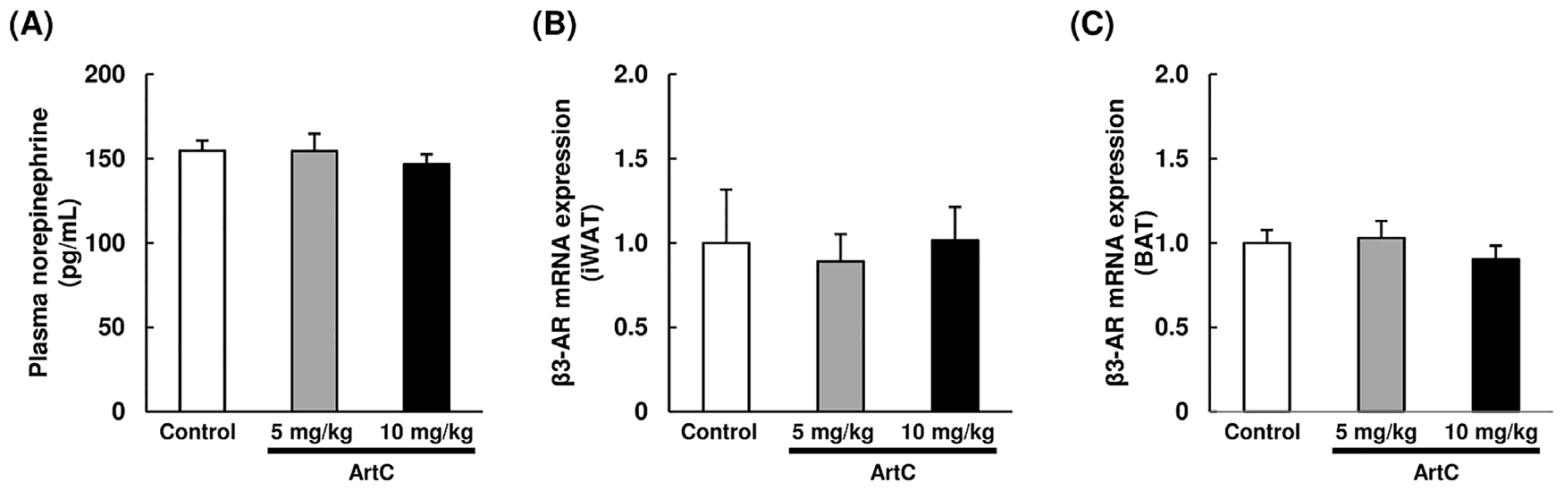
Plasma NE concentrartion and mRNA levels of β3-AR in iWAT and BAT of mice treated with vehicle (control) or ArtC (5 mg/kg or 10 mg/kg) for 4 weeks. (A) Plasma NE concentrartion (B) mRNA levels of β3-AR in iWAT, (C) mRNA levels of β3-AR in BAT. The mRNA levels are expressed as fold of control (= 1) after normalization using the expression levels of the TBP gene. Data are shown as means ± SEM (*n* = 10).

As expected, the results obtained from C3H10T1/2 cells and primary iWAT-derived adipocyte studies demonstrated that administration of ArtC induced brown-like adipocyte formation in mice. To confirm whether oral administration of ArtC induces brown-like adipocyte formation in mice, ArtC was administered to mice for 4 weeks, then UCP1 protein expressions in iWAT, eWAT, and BAT were examined using immunohistochemistry and immunoblotting. Body weight gain, food intake, and adipose tissues weight (iWAT, eWAT, and BAT) were not affected by the administration of ArtC (S1 Table). The H&E staining of iWAT obtained from the ArtC groups clearly showed multilocular adipocytes which imply that the morphological characteristic of brown-like adipocytes vary in a dose-dependent manner (Fig 5A). Moreover, these multiocular adipocytes showed distinct immunostaining of UCP1 and PRDM16 (Fig 5A). By contrast, multilocular adipocytes and UCP1-immunopositive cells were not observed in eWAT of the ArtC groups (Fig 5B). H&E staining and UCP1 and PRDM16 immunostaining did not differ in BAT from the three groups (Fig 5C). To further confirm the results obtained from immunohistochemistry and studies of mitochondrial function, the expression levels of UCP1 and CoxIV protein were examined in iWAT and BAT. The expression levels of UCP1 and CoxIV protein were significantly elevated in iWAT of mice administered 10 mg/kg ArtC compared to the control group (Fig 6A). However, the expression levels in BAT of both UCP1 and CoxIV did not differ among the three groups (Fig 6B). Further, the plasma NE concentration and β3-AR mRNA level in iWAT and BAT were not significantly affected by the administration of ArtC (Fig 7).

## Discussion

Brown-like adipocytes can develop in WAT in response to various stimuli, such as cold exposure and the presence of β3-AR agonists [29]. The activities of brown-like adipocytes reduce body fat accumulation via induction of thermogenesis associated with UCP1, and result in reduction in the risk of metabolic diseases [1]. These findings indicate that a new strategy of induction of brown-like adipocytes in WAT may be one of the most plausible ways of preventing and treating obesity and diabetes. Therefore, considerable attention has been focused on dietary factors or other natural compounds that may be beneficial in the induction of brown-like adipocyte proliferation. However, there has been little evidence that diet-derived factors can directly induce browning of the white adipocytes which are responsible for anti-obesity via thermogenesis. In addition, the underlying mechanism of induction of brown-like adipocytes by diet-derived factors has been unclear. Therefore, we screened various dietary compounds and found that ArtC was a potential inducer of brown-like adipocytes.

First, we demonstrated that ArtC significantly induced brown-like adipocyte formation using two cellular systems (C3H10T1/2 cells and primary iWAT-derived adipocytes), conferring the ability to enhance thermogenesis. ArtC is a prennyl cinammic acid derivative, and the principal component of Brazilian propolis [16, 17]. It has been reported that ArtC has multiple biological functions such as neuroprotective [30] and anti-angiogenic effects [31] and inhibition of the release of cys-leukotrienes [32]. Previously we found that ArtC is a potent PPARγ agonist and significantly inhibits the downregulation of adiponectin expression in 3T3-L1 adipocytes [23]. These results suggest that ArtC, like rosiglitazone, induced brown-like adipocytes via activation of PPARγ [13, 27]. In the present study, we demonstrated that the administration of ArtC to C3H10T1/2 cells resulted in significant induction of UCP1 and PRDM16 protein; however, administration of a PPARγ antagonist (T0070907) significantly inhibited the expression of these proteins. The present results also showed that ArtC-treated adipocytes with co-administration of cycloheximide significantly inhibited the degradation of PRDM16 protein and extended the half-life. Therefore, these results clearly demonstrated that ArtC induced brown-like adipocyte formation via its action as a PPARγ agonist and stabilization of PRDM16. These findings led us to question whether administration of ArtC would significantly induce brown-like adipocyte formation in *in vivo* systems. Our data clearly demonstrated that the oral administration of ArtC (10 mg/kg) significantly caused the formation of brown-like adipocytes and induced the expression of UCP1, PRDM16, and CoxIV proteins in iWAT of mice. This is the first study to show that orally administered ArtC induces brown-like adipocytes *in vitro* and *in vivo*, demonstrating an additional potential mechanism of brown-like adipocyte formation. We are planning to examine whether ArtC induced thermogenesis by monitoring adipose tissue and rectal temperature in mice.

It is also possible that brown-like adipocyte formation is induced by the β3-adrenergic signaling pathway via the sympathetic nervous system (SNS) [33]. For example, fish oil intake induces UCP1 expression in iWAT and BAT via the SNS accompanied by an increase in urine NE level [34]. In the present study, the plasma NE concentration and the mRNA level of β3-AR in iWAT and BAT did not differ between the groups. These results suggest that induction of brown-like adipocytes by ArtC is not due to activation of the β3-adrenergic signaling pathway via the SNS.

The present study raises another question, why did ArtC not significantly induce UCP1 expression in BAT? It is known that BAT is more sensitive and dominant than WAT for activation of the β3-adrenergic signaling pathway via the SNS [35, 36]. As shown in this study, ArtC did not significantly affect β3-adrenergic signaling. Although the exact mechanism remains unclear, the different sensitivity of the β3-adrenergic signaling pathway in BAT and WAT may have a role in the different response of UCP1 induction.

In conclusion, we have demonstrated that ArtC significantly induces brown-like adipocytes, and this significant induction is due to activation of PPARγ and stabilization of PRDM16 protein. These findings may provide insight into the browning of white adipocytes, including the molecular mechanism mediated by diet-derived factors, and demonstrate a novel biological function of ArtC with regard to increasing energy expenditure by browning of white adipocyte formation.

## Supporting Information

S1 Table Body weight, food intake, and relative tissue weights in C57BL/6J mice orally administered vehicle or ArtC for 4 weeks. (PDF)
