# Supplementary material for: Artepillin C, a Typical Brazilian Propolis-Derived Component, Induces Brown-Like Adipocyte Formation in C3H10T1/2 Cells, Primary Inguinal White Adipose Tissue-Derived Adipocytes, and Mice
Source: PLoS One. 2016 Sep 6;11(9):e0162512. doi: 10.1371/journal.pone.0162512 (PMC5012562; doi:10.1371/journal.pone.0162512)
Supplement: S1 Table — (PDF) [file pone.0162512.s001.pdf]

S1 Table. Body weight, food intake, and relative tissue weights in C57BL/6J mice orally administered vehicle or ArtC for 4 weeks.[1]

	Control	ArtC (5 mg/kg)	ArtC (10 mg/kg)
**Initial body weight, *g***	**18.6 ± 0.50**	**18.6 ± 0.28**	**18.5 ± 0.25**
**Final body weight, *g***	**22.9 ± 0.41**	**22.7 ± 0.25**	**22.9 ± 0.30**
**Food intake, *g/(4 weeks・mouse)***	**151.4 ± 6.0**	**146.1 ± 5.9**	**155.0 ± 7.8**
**Epididymal WAT, *g/100 g body***	**1.61 ± 0.06**	**1.56 ± 0.06**	**1.61 ± 0.07**
**Inguinal WAT, *g/100 g body***	**0.88 ± 0.06**	**0.87 ± 0.05**	**1.00 ± 0.06**
**Interscapular BAT, *g/100 g body***	**0.49 ± 0.02**	**0.45 ± 0.03**	**0.54 ± 0.03**

[1] **Values are means ± SEM, *n* =10.**
